# Physiologically-Based Pharmacokinetic Modeling and In Vitro–In Vivo Correlation of TV-46000 (Risperidone LAI): Prediction from Dog to Human

**DOI:** 10.3390/pharmaceutics16070896

**Published:** 2024-07-04

**Authors:** David Bibi, Raphael Bilgraer, Lilach Steiner, Hussein Hallak

**Affiliations:** Non-Clinical Development, Teva Pharmaceutical Industries Ltd., Netanya 4250400, Israel

**Keywords:** physiologically-based pharmacokinetic modeling (PBPK), in vitro–in vivo correlation (IVIVC), long-acting injectable (LAI), TV-46000, risperidone, schizophrenia

## Abstract

The interest in the development and therapeutic application of long-acting injectable products for chronic or long-term treatments has experienced exponential growth in recent decades. TV-46000 (Uzedy, Teva) is a long-acting subcutaneous (sc) injectable formulation of risperidone, approved for the treatment of schizophrenia in adults. Following sc injection, the copolymers together with risperidone precipitate to form a sc depot under the skin to deliver therapeutic levels of risperidone over a prolonged period of either 1 month or 2 months, depending upon the dose. This work presents the strategy and the results of the physiologically-based pharmacokinetic (PBPK) modeling and establishing of in vitro–in vivo correlation (IVIVC) for the prediction of TV-46000 pharmacokinetic profile in humans, using in vitro release, intravenous (iv), and sc single-dose pharmacokinetic data in beagle dogs. The resulting simulated TV-46000 PK profile in humans showed that the shape of the predicted risperidone and its active metabolite 9-OH-risperidone PK profiles was different from the observed one, thus suggesting that the TV-46000 release profile was species-dependent and cannot be directly extrapolated from dog to human. In conclusion, while level A IVIVC cannot be claimed, this work combining PBPK and IVIVC modeling represents an interesting alternative approach for complex injectable formulations where classical methods are not applicable.

## 1. Introduction

Long-acting injectable (LAI) products are concentrated formulations designed to release drugs slowly over time after intramuscular or subcutaneous injection. In recent decades, there has been a remarkable increase in interest in the development and therapeutic utilization of LAI products for chronic or long-term treatments, especially within the antipsychotic field [1,2,3,4,5]. LAI antipsychotics products offer various clinical advantages over conventional oral therapies, including reduced dose frequency, improved treatment adherence, avoidance of first-pass metabolism, extended apparent body half-life, and enhanced control of the clinical response by lowering the probability of rebound symptoms or rapid relapse, contributing to an overall improvement in the quality of life [6]. TV-46000 (Uzedy, Teva) is an FDA-approved long-acting subcutaneous injectable (LAI) formulation of risperidone, a dopamine type 2 and serotonin type 2 receptor antagonist [7] used as treatment for schizophrenia in adults [8]. The TV-46000 formulation of risperidone is composed of biodegradable copolymers and organic solvent (DMSO) and uses a novel drug delivery technology to ensure sustained concentrations of risperidone [9]. Following subcutaneous injection, an in situ depot forms under the skin as solvent is exchanged between DMSO in the formulation and water in the biological fluid. In the in situ depot, risperidone is entrapped to ensure low initial bursts and avoid uncontrolled release (data on file Teva). TV-46000 is administered via a single sc injection using a once monthly (Q1M) or once every two months (Q2M) dosing schedule and is designed to provide a more convenient and less painful way to administer risperidone. In the phase 3 risperidone subcutaneous extended-release (RISE) study in patients with schizophrenia, two treatment stages were conducted. The first stage consisted of a 12-week, open-label stabilization phase with oral risperidone, followed by an open-ended, randomized, double-blind, placebo-controlled, relapse-prevention phase with subcutaneous TV-46000. This study was conducted at 69 clinical sites across the USA and Bulgaria. TV-46000 significantly increased time to impending relapse compared to placebo, with a 5-fold increase in the Q1M group and a 2.7-fold increase in the Q2M group [8]. 

PBPK models have gained significant prominence in drug discovery and development, as well as in regulatory submissions and reviews. This unique tool enables the estimation of a compound’s pharmacokinetic (PK) profile using preclinical absorption, distribution, metabolism, and excretion (ADME) data. PBPK models provide a means to assess exposure in a target organ following drug administration by considering the absorption and disposition rates in that organ, along with any applicable metabolism within it. Leveraging PK data from a specific dose schedule, PBPK models facilitate the evaluation of PK profiles for various dose schedules and/or administration routes [10]. Another predicting mathematical model is in vitro–in vivo correlation (IVIVC), which elucidates the connection between in vitro drug dissolution/release and the in vivo response of various formulations.

The development of an IVIVC allows the prediction of in vivo drug properties from its in vitro release profile, potentially serving as a substitute for bioequivalence studies and thereby reducing regulatory burdens. In vitro dissolution tests that are relevant to in vivo conditions offer significant advantages, such as lowering the cost of in vivo studies and accelerating the drug development process. Consequently, exploring IVIVC has become a crucial aspect of developing long-acting injectable (LAI) formulations. Unfortunately, no specific regulatory guidelines for IVIVC in LAI pharmaceuticals are available at the time of this writing. Currently, the principles designed for oral extended-release drug products are being applied to parenteral LAIs. Despite the creation of various in vitro dissolution methods to characterize drug release from LAIs, there are still only a limited number of reported IVIVC cases for neuroleptics in the literature [11].

The first objective of this work was to develop a PBPK model to predict risperidone and its active metabolite, 9-OH-risperidone plasma PK profile, following TV-46000 sc administration. The PBPK model was first calibrated in dogs and then extrapolated to humans. With the available human data, a multi-step approach was employed. This involved leveraging the benefits of PBPK modeling for mechanistic deconvolution, utilizing the Weibull release function. The second objective was to establish an in vitro–in vivo correlation (IVIVC) in humans. This correlation allowed the prediction of PK profiles for new dose levels based on their in vitro release profile, thus providing a means to avoid actual in vivo testing by defining the design space and identifying the critical quality attributes that must be controlled (directly or indirectly) to ensure that the final therapeutic product consistently meets safety, efficacy, stability, and performance standards.

## 2. Materials and Methods

### 2.1. Data

#### 2.1.1. In Vitro Release Data

An accelerated in vitro method was developed to assess the in vitro release rate of TV-46000 (batch GE7305) at two different doses (50 mg and 225 mg). The TV-46000 formulation was injected in a 200 mL medium at pH 6.0 under agitation (170 rpm). Risperidone cumulative amount was assessed at 16 time points (2, 6, 12, 24, 30, 48, 72, 96, 168, 192, 216, 264, 336, 408, 504, and 672 h). The experiment was performed in 12 replicates of the clinical batch (batch GE7305) and the average risperidone cumulative amount at each time point was computed.

#### 2.1.2. In Vitro Metabolism Data

To characterize the metabolism of risperidone, an in vitro phenotyping assay was conducted using human recombinant cytochromes P450 (rCYP) 2D6 and 3A4. The formation of risperidone active metabolite, 9-OH-risperidone, was measured over time, and the Michaelis–Menten constants K_m_ and V_m_ were calculated for each rCYP.

#### 2.1.3. Dog Data

Two studies in dogs were conducted to characterize the PK profiles of risperidone and 9-OH-risperidone. In the first study, male beagle dogs (n = 6) were administered a formulation of risperidone via intravenous (iv) infusions during period 1 and via immediate-release (IR) sc injections during period 2. The formulation of risperidone was produced by the study sponsor, Teva Pharmaceuticals in the Teva PLIVA production plant, Zagreb, Croatia. For the quantification of risperidone and 9-OH-risperidone in dog plasma, a qualified LC-MS/MS method was employed. For PK evaluation purposes, blood was collected from all animals as follows: period 1: pre-dose, 5, 15, 30 min, 1, 2, 4, 6, 8, 10, 12, 18, 24, 36, 48, and 72 h post-dose; period 2: pre-dose, 5, 15, 30 min, 1, 2, 4, 6, 8, 10, 12, 18, 24, 36, 48, 60, 72, 96, and 120 h post-dose. In the second study, three groups of 5 male and 5 female beagle dogs received repeated administrations of TV-46000 doses of 7, 20, or 50 mg/kg, respectively, once a month for up to 3 months, followed by a 12-week treatment-free recovery period. For pharmacokinetic evaluation purposes, blood was collected from all animals, as follows: pre-dose, 1, 3, 6, 24, 168, 336, and 672 h post-dose. 

#### 2.1.4. Human Data

A Phase 1 sequential open-label single ascending dose (SAD) and multiple-ascending dose (MAD) study was conducted in patients with a diagnosis of schizophrenia to evaluate the safety, tolerability, and pharmacokinetics (PK) of TV-46000 and is described elsewhere [12]. Study participants were clinically stable patients who were not currently on antipsychotic treatment other than oral risperidone. Study participants provided written informed consent before any protocol-related procedures commenced and were screened against the inclusion and exclusion criteria. Those who met the eligibility criteria were sequentially assigned to 1 of 8 cohorts (n = 12 for cohorts 1–7 and n = 15 for cohort 8) and received administrations of oral risperidone (ZyGenerics, Pennington, NJ, USA) and sc injections of TV-46000 as detailed below. The oral and sc injection dosing regimens for each cohort were as follows, respectively: cohort 1, 2 mg/day orally and 50 mg/month given as a single sc dose; cohort 2, 2 mg/day orally and 75 mg/month given as a single sc dose; cohort 3, 3 mg/day orally and 100 mg/month given as a single sc dose; cohort 4, 4 mg/day orally and 150 mg/month given as a single sc dose; cohort 5, 6 mg/day orally and 225 mg/month given as a single sc dose; cohort 6, 3 mg/day orally and 75 mg/month given 3 times as sc dose; cohort 7, 5 mg/day orally and 150 mg/month given 3 times as an sc dose; and cohort 8, 5 mg/day orally and 225 mg/month given as a single sc dose. The sc injections were administered in the abdomen for cohorts 1 through 7 and in the upper arm for cohort 8. The study consisted of a screening period of up to 26 days; an inpatient oral risperidone tolerability testing period of 7 days, followed by a 7-day inpatient washout period; an inpatient and outpatient TV-46000 treatment period of up to approximately 4 weeks (cohorts 1 and 2) or up to 12 weeks (cohorts 3 through 8); and a follow-up period of approximately 8 weeks (cohorts 1, 2, 6, and 7) or 4 weeks (cohorts 3, 4, 5, and 8) that included PK blood sampling and safety assessments. For PK evaluation purposes, blood was collected from all subjects as follows: cohort 1–2: pre-dose, 0.5, 1, 2, 3, 4, 6, 8, 12, 24, 30, and 36 h post-dose, and on days 3, 4, 5, 6, 7, 8, 10, 12, 15, 18, 22, 25, 29, 36, 43, 50, 57, 64, 71, 78, and 84 post-dose; cohort 3–5 and 8: pre-dose, 0.5, 1, 2, 3, 4, 6, 8, 12, 24, 30, and 36 h post-dose, and on days 3, 4, 5, 6, 7, 8, 10, 12, 15, 18, 22, 25, 29, 36, 43, 50, 57, 64, 71, 78, 85, 92, 99, 106, and 112 post-dose.

### 2.2. Physiologically-Based Pharmacokinetic Modeling and Simulation

#### 2.2.1. Modeling Strategy

**Figure 1:**
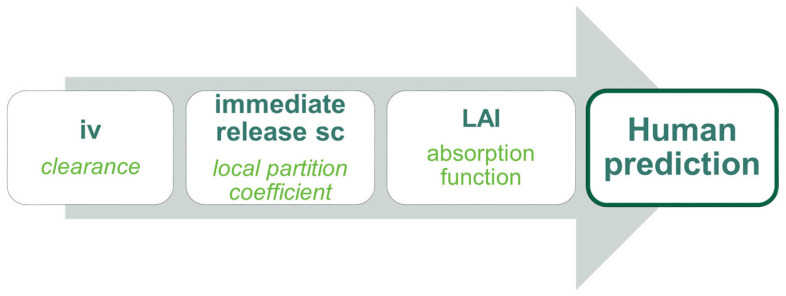
Flowchart of the general modeling approach for FIH prediction.

The overall modeling strategy for human PK prediction consisted of building and calibrating a PBPK model in dogs using available experimental data and extrapolating it to human physiology (Figure 1). To build a physiologically relevant model for TV-46000 in dogs, it was necessary to characterize the essential PK parameters that are driving the overall kinetics. Clearance was the first key parameter that needed to be characterized from an immediate-release route of administration (ideally iv) to overcome the flip-flop kinetic phenomenon that occurs with sustained release formulations [13]. The clearance was characterized using the data generated following iv administration to dogs. Once the clearance was properly defined in the iv model, the tissue-to-plasma partition coefficient (K_p_) for the subcutaneous tissue (i.e., adipose) was the second essential parameter that was required to describe the distribution kinetic of risperidone, independently of the release kinetics from the formulation. The data generated from the IR sc administration in dogs was used for that purpose. The last step of the modeling work was to characterize the release kinetic of the long-acting formulation of risperidone (TV-46000). For that purpose, the previously defined parameters were fixed in a long-acting injectable (LAI) model and a Weibull release function was fitted to the dog experimental LAI sc data. To extrapolate from dog to human, assumptions had to be made around some of the model parameters. In the PBPK model, since Kp and the drug release function for humans are unknown, these assumptions are necessary. Weibull function is related to the release kinetics, while Kp is a physiological parameter reflecting the absorption of the active pharmaceutical ingredient into the tissues. Based on several preclinical studies (data on file, Teva and the Risperidal NDA [14]), the dog was chosen as the most relevant model to humans for testing risperidone absorption following TV-46000 sc administration. Thus, the K_p_ for the subcutaneous tissue as well as the Weibull release function were assumed to be preserved in humans. Since the major metabolite of risperidone (9-OH-risperidone) is active, it had to be included in the final human prediction. For that purpose, clearance in humans was characterized using in vitro metabolism data to generate the kinetics of metabolite formation (metabolite tracking). All other physiological parameters were automatically adjusted from dogs to humans by GastroPlus™ software. 

#### 2.2.2. Physicochemical Properties

The main physicochemical properties used in the initial PBPK model for risperidone and 9-OH-risperidone were obtained from the literature [15,16] (Table 1).

#### 2.2.3. Intravenous Model in Dogs

Geometric mean and coefficient of variation (CV) values were computed from individual dog PK data following a single iv administration of risperidone in dogs at 1.0 mg/dog. The group geometric mean data were modeled using a compartmental PK approach, and the optimization module was used to identify PK values that gave the best possible fit of the experimental data. Physiological parameters were automatically scaled to the selected dog physiology by GastroPlus software, without any manipulation. The following PK parameters were selected for optimization: total clearance from central compartment (CL), volume of distribution (Vc), rate constant for transfer from central to peripheral compartment (K12), and rate constant for transfer from peripheral to central compartment (K21).

#### 2.2.4. Immediate-Release sc Model in Dogs

Geometric mean and CV values were computed from individual PK data following single sc administration of IR risperidone in dogs at 5.0 mg/dog. The group geometric mean data were modeled using a PBPK approach with the relevant physiology (beagle dog, fasted, average body weight = 10 kg) and the previously obtained clearance value from the iv model. In dogs, the sc PBPK models were generated by GastroPlus software without any manipulation. In the model, the Weilbull function parameters and K_p_ were optimized, specifically the optimization module identified the K_p_ for the adipose tissue (relevant to the sc route) that gave the best fit of the experimental data. 

#### 2.2.5. Long-Acting Injectable sc Model in Dogs

Combining the knowledge obtained from the previously calibrated iv (clearance) and IR sc (K_p_) models, the group geometric mean data were modeled using a PBPK approach with relevant physiology (beagle dog, fasted, average body weight = 10 kg). The PBPK model was generated by GastroPlus and the optimization module was used to identify parameters of the Weibull release (Equation (1)) that gave the best possible fit of the experimental data. The following Weibull function parameters were selected for optimization: the percentage of maximum release, time lag, total released, Phase 1 fraction, Phase 1 time scale, Phase 1 shape, Phase 2 time scale, and Phase 2 shape. 

Equation (1): The double-Weibull function.

(1) $$\%DoseReleased=Max\times \left(1-f_{1}e^{\left[\frac{{-\left(t-Tlag\right)}^{b_{1}})}{A_{1}}\right]}-f_{2}e^{\left[\frac{{-\left(t-Tlag\right)}^{b_{2}})}{A_{2}}\right]}\right)$$

where *Max* is the percentage of maximum release, *Tlag* is the lag time, *f*_1_ is the fraction released during phase 1, *f*_2_ is the fraction released during phase 2, *A*_1_ is the time scale factor for phase 1, *A*_2_ is the time scale factor for phase 2, *b*_1_ is the shape for phase 1, and *b*_2_ is the shape for phase 2.

#### 2.2.6. Physiologically-Based Pharmacokinetic Model for TV-46000 in Humans

As the first objective of the study was to predict the human PK profile based on dog PK data and without any human data, several assumptions were made. Specifically, it is assumed that the Kp and the drug release function were consistent between species. Thus, the K_p_ for the adipose tissue and the double-Weibull release function were obtained from the PBPK models in dogs and used in the human model. The risperidone clearance in humans, the human blood-to-plasma concentration ratio, and the fraction unbound (f_u_) in plasma were extracted from the literature [16]. All other physiological parameters were automatically scaled to the selected human physiology by GastroPlus software.

### 2.3. In Vitro–In Vivo Correlation

#### 2.3.1. Mechanistic Deconvolution

Mechanistic deconvolution was performed by fitting a PBPK model with a triple Weibull absorption function to the observed human PK profile of each dose strength (i.e., 50 and 225 mg). These specific doses were chosen to cover the entire range of tested doses for TV-46000, aligning with the approved clinical dosage range. The obtained Weibull parameters were used to compute the actual percentage of dose released (% released) at each observed time point according to Equation (2). 

Equation (2): The triple-Weibull function used for deconvolution.

(2) $$\%DoseReleased=Max\times \left(1-f_{1}e^{\left[\frac{{-\left(t-Tlag\right)}^{b_{1}})}{A_{1}}\right]}-f_{2}e^{\left[\frac{{-\left(t-Tlag\right)}^{b_{2}})}{A_{2}}\right]}-f_{3}e^{\left[\frac{{-\left(t-Tlag\right)}^{b_{3}})}{A_{3}}\right]}\right)$$

where *Max* is the percentage of maximum release, *T_lag_* is the lag time, *f*_1_ is the fraction released during phase 1, *f*_2_ is the fraction released during phase 2, *f*_3_ is the fraction released during phase 3, *A*_1_ is the time scale factor for phase 1, *A*_2_ is the time scale factor for phase 2, *A*_3_ is the time scale factor for phase 3, *b*_1_ is the shape for phase 1, *b*_2_ is the shape for phase 2, and *b*_3_ is the shape for phase 3.

#### 2.3.2. In Vitro Time Scaling 

Phoenix^®^ WinNonlin^®^ version 8.1 IVIVC Toolkit software (Certara, Princeton, NJ, USA) was used to compare in vitro dissolution and in vivo deconvoluted data as time-versus-time plots at matched values for absorption and release. In vivo versus in vitro times at each fraction absorbed were obtained by linear interpolation and presented as a Levy plot. A linear regression model was fitted to the data points with an intersection at the origin and the corresponding equation was used for time scaling of the in vitro time points.

#### 2.3.3. Correlation

Following time scaling, in vitro percent released were plotted against in vivo (deconvoluted) percent absorbed at each shared time point in order to fit a regression function and establish the IVIVC model. 

#### 2.3.4. Convolution and Model Assessment

To assess the IVIVC model, internal prediction was performed using the modeled data for each dose strength. The time points from the in vitro accelerated profiles were scaled using the obtained time scaling factor, then the in vitro percent released was converted to the predicted in vivo percent absorbed using the established IVIVC model. The resulting percent absorbed profiles were used as input in the human risperidone PBPK model used for deconvolution to simulate the TV-46000 PK profile in humans. Exposure parameters (C_max_, AUC_0-t_) were computed from the simulated profiles and compared with the observed ones to compute the percent prediction errors (%PE). 

### 2.4. Software

All the presented PBPK modeling work was performed using the GastroPlus™ software version 9.6 (Simulations Plus, Inc., Lancaster, CA, USA) and the additional dosage route module with the transdermal option. The optimization module was used to search for values of certain input parameters that yield simulation outputs that fit the observed data. The metabolite tracking was performed using the metabolism and transporters module. The levy plot for the time scaling of in vitro release profiles was obtained using Phoenix IVIVC Toolkit version 2.1 (Certara L.P.). Data preprocessing and descriptive statistics were performed using R version 3.6.1.

## 3. Results

### 3.1. In Vitro Release and Metabolism Data

**Figure 2:**
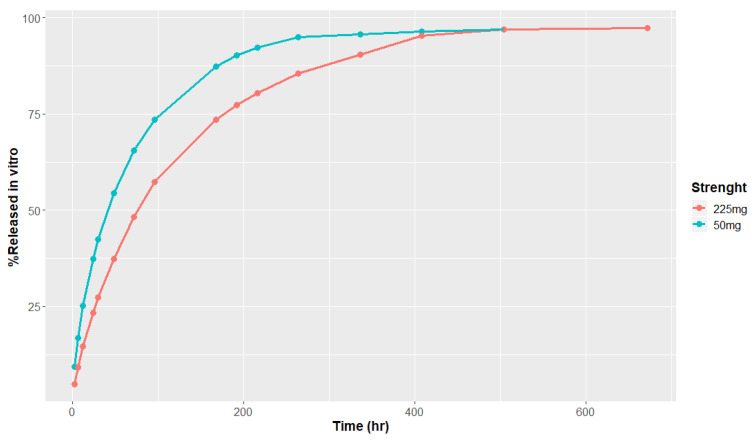
In vitro accelerated release profiles (pH 6.0) of TV-46000.

The in vitro release rate of risperidone from TV-46000 was assessed at two different dose strengths, 50 mg and 225 mg. The average risperidone cumulative amount at each time point was computed and presented in Figure 2. An in vitro phenotyping assay was conducted using human recombinant cytochromes P450 (rCYP) 2D6 and 3A4 to measure risperidone Michaelis–Menten constants (Table 2). 

### 3.2. In Vivo Models to Characterize Risperidone Pharmacokinetics following Different Routes of Administration in Dogs

**Figure 3:**
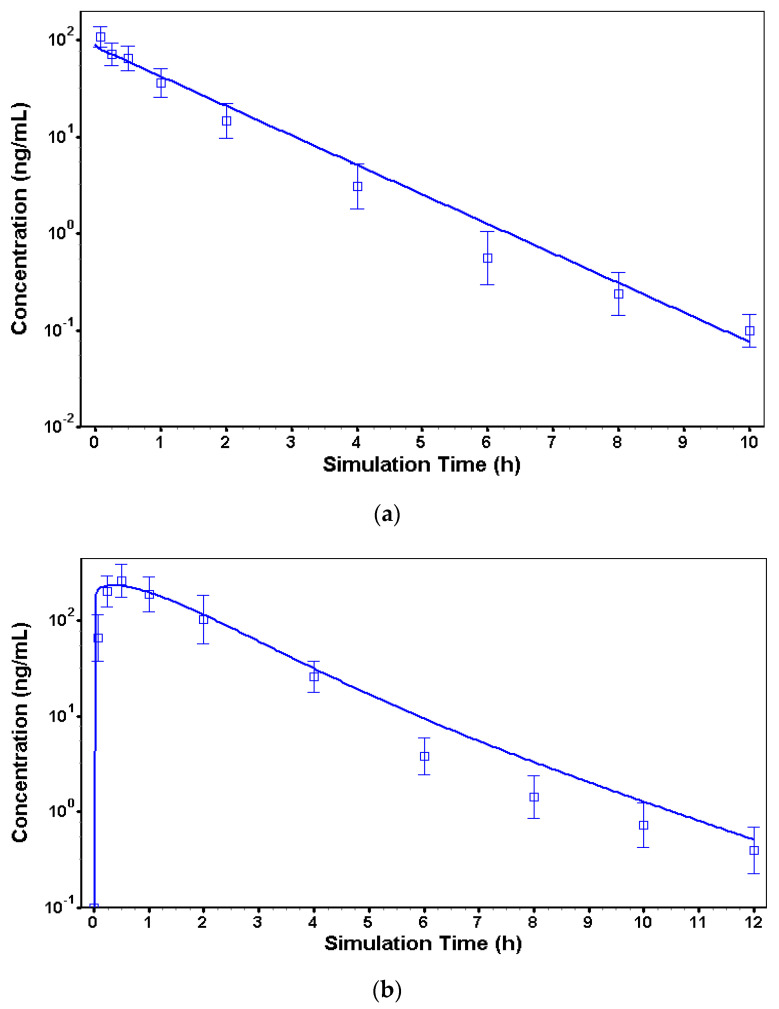

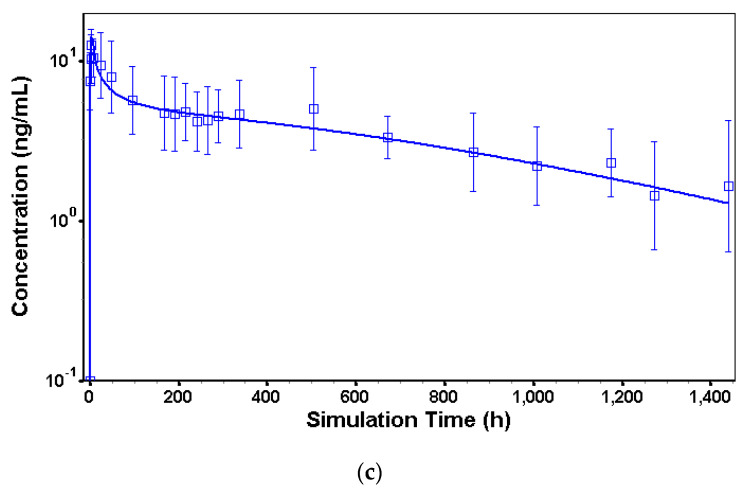
Plasma concentrations versus time-predicted profile for risperidone following different routes of administration in dogs. (**a**) Plasma concentration versus time predicted profile (line) and experimental data (squares) for risperidone single iv administration at 1 mg in dogs (y-log10 scale). (**b**) Plasma concentration versus time predicted profile (line) and experimental data (square) for risperidone single IR sc administration at 5 mg in dogs (y-log10 scale). (**c**) Risperidone plasma concentration versus time predicted profile (line) and experimental data (square) following single sc administration of TV-46000 Grade 4-2 at 50 mg in dogs (y-log10 scale). Legend: Plasma concentration versus time predicted profile (line) and experimental data (squares) for risperidone (**a**) single iv administration of risperidone at 1.0 mg in dog; (**b**) single immediate release risperidone sc administration at 5.0 mg in dog; (**c**) following single sc administration of TV-46000 at 50 mg in dog. All y-log10 scale.

To characterize the essential pharmacokinetic parameters driving the kinetic release of risperidone, three in vivo models of the pharmacokinetics of risperidone in dogs were constructed. In the iv model, risperidone plasma protein binding parameters in dogs from the literature [16], blood-to-plasma ratio, 0.51, and fraction unbound, 8.3%, were implemented in the initial PBPK model. The iv data was then used to characterize the terminal elimination phase of risperidone in dogs. Compartmental PK modeling was used to describe the iv data and extract the PK parameters; the optimized values for clearance (CL) was 8.3 L/h with a volume of clearance (Vc) of 1.13 L/kg, and the elimination rate constants K12 and K21 were zero. The best fit was obtained with a 1-compartment PK model (R^2^ = 0.91). The simulated PK profile using the calibrated iv model is presented in Figure 3a. The exposure parameters (C_max_, AUC_0–∞_) were computed from the simulated profile and compared to the observed values from the experimental data (Table 3).

Prior to the optimization of the K_p_, the previously calibrated iv model was switched to the sc route of administration and the PBPK model option was selected. The physiology was set to fasted beagle dog with a body weight of 10 kg (as per experimental conditions), and the clearance was fixed to the optimized iv clearance. The K_p_ value for the adipose tissue that gave the best fit with the experimental data was 1.9 for risperidone administered as an IR sc injection of 5.0 mg per dog. The simulated PK profile using the calibrated IR sc model is shown in Figure 3b. The exposure parameters (C_max_, AUC_0–∞_) were computed from the simulated profile and compared to the observed values from the experimental data (Table 3). 

The last step of the modeling work was to characterize the release kinetic of the TV-46000. For that purpose, the previously defined parameters were fixed in a long-acting injectable (LAI) model and a Weibull release function was fitted to the dog experimental LAI sc data. The fitted double-Weibull parameters are shown in Table 4. The double-Weibull function described well the experimental data as shown in Figure 3c. The exposure parameters (C_max_, AUC_0–∞_) were computed from the simulated profile and compared to the observed values from the experimental data (Table 3). 

### 3.3. In Vivo Model in Humans

**Figure 4:**
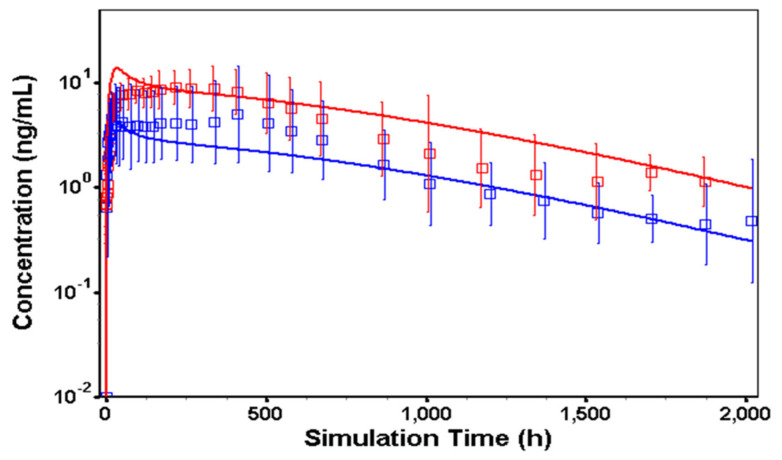
Observed (squares and error bars) vs. predicted risperidone and 9-OH risperidone concentration following TV-46000 long acting sc injectable 50 mg in humans. Legend: Observed (squares and error bars) versus predicted risperidone (blue) and 9-OH-risperidone (red) plasma concentration versus time profile for TV-46000 single sc administration of 50 mg in humans.

Similar to the model development in dogs, three key parameters (i.e., clearance, K_p_, and the drug release function) were essential to build a human PBPK model for TV-46000. As one of our objectives in this study was to predict the human pk profile based on dog data, a naïve approach was used to simulate a scenario where no human data was available for the model development. With the lack of data to characterize the K_p_ (IR sc data) and the drug release function in humans, assumptions needed to be made around those parameters. For the clearance, the in vitro metabolism values from Table 2 were used. Values for the blood-to-plasma (B/P) concentration ratio, 0.67 and the fraction unbound in humans, 10% were extracted from the literature [16]. The K_p_ and the drug release function were assumed to be similar in dogs and in humans, and values obtained during the dog model calibration were used in the human PBPK model. These assumptions seem to be reasonable knowing that the dog is considered as a clinically relevant model for subcutaneous administration. For the 9-OH-risperidone metabolite, total clearance was obtained from the literature (CL = 80 mL/min) [17]. All other physiological parameters were automatically scaled to the selected human physiology (American, healthy, fasted, 30 years old, 65 kg, 176.4 cm). The simulated TV-46000 PK profile using the human PBPK model is shown in Figure 4, and the corresponding risperidone mean exposure parameters (C_max_, AUC_0–∞_) are shown in Table 5. 

### 3.4. In Vivo–In Vitro Correlation Modeling

#### 3.4.1. Deconvolution

**Figure 5:**
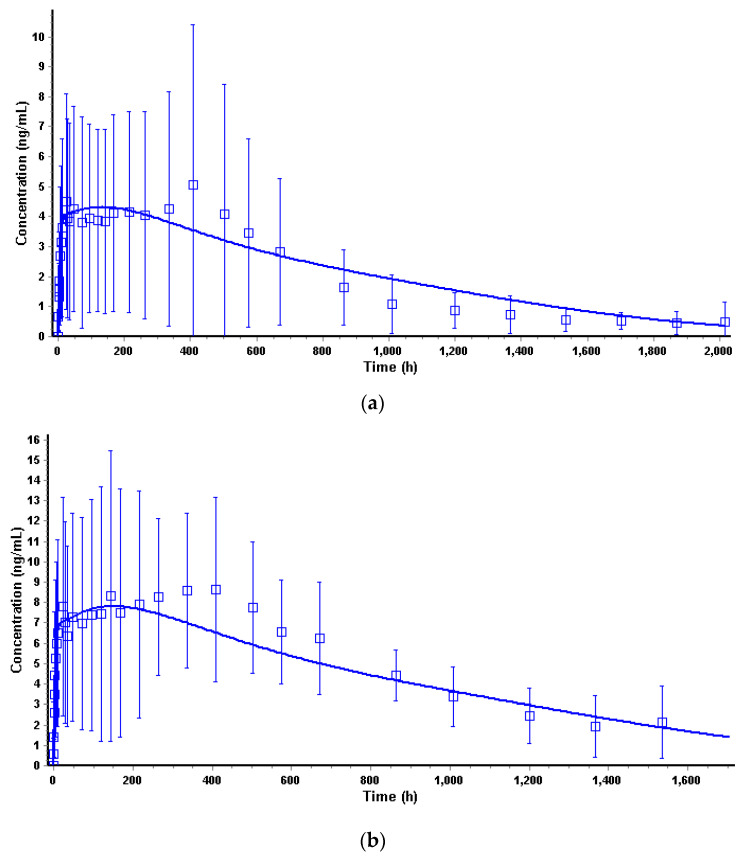

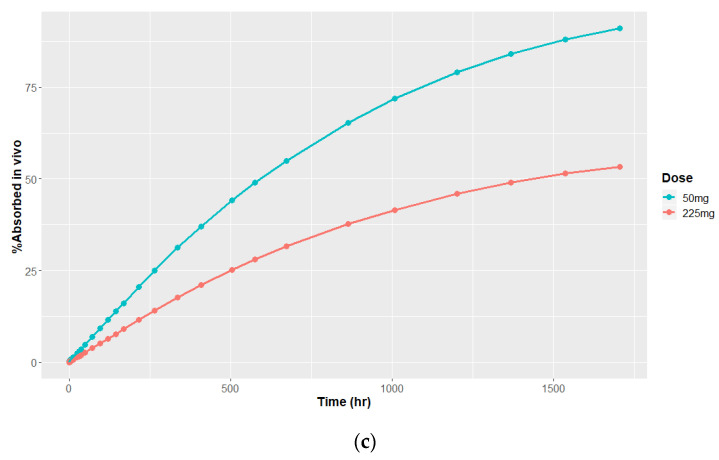
Simulated versus observed TV-46000 convoluted PK profile in humans. (**a**) Simulated versus observed TV-46000 PK profile in humans at 50 mg. (**b**) Simulated versus observed TV-46000 PK profile in humans at 225 mg. (**c**) In vivo percent absorbed versus time for TV-46000 in humans at 50 and 225 mg obtained by deconvolution.

The first step of the IVIVC model building was to generate the deconvoluted profiles from the in vivo PK profiles. The PBPK-based deconvolution approach is an alternative to the traditional deconvolution methods (Wagner–Nelson, Loo–Riegelman) that enables the incorporation of physiological processes to provide more flexibility in establishing IVIVC for complex formulations [18]. As described earlier, the human PBPK model for TV-46000 sc administration was translated from dogs using the specific human parameters extracted from the literature (B/P ratio, fraction unbound), as well as the generated in vitro metabolism constants. A dedicated Weibull release function was fitted to the experimental PK profile of each dose level (Figure 5). The exposure parameters (C_max_, AUC_0–∞_) were computed from the simulated profiles and compared to the observed values from the experimental data (Table 6). The fitted Weibull parameters shown in Table 7 were used to perform the actual deconvolution according to Equation (2). 

#### 3.4.2. Time Scaling and Correlation

**Figure 6:**
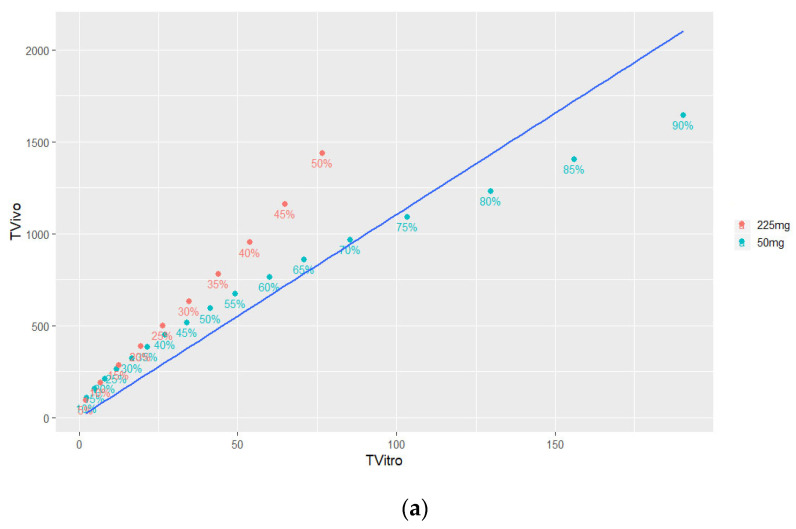

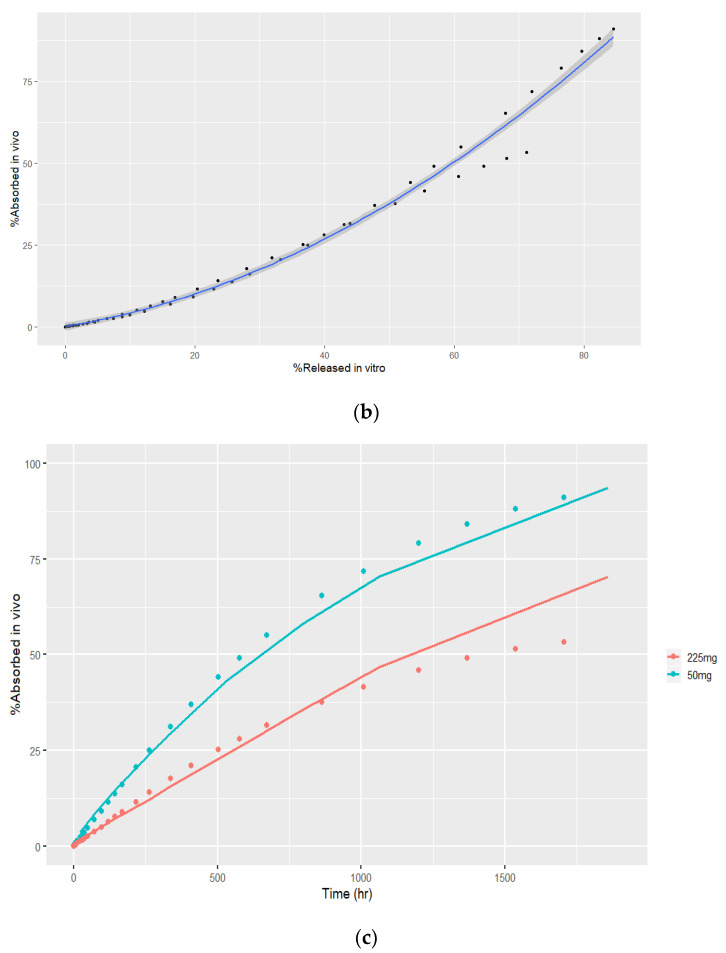
Time scaling of in vitro release data and correlation to deconvoluted in vivo pk profiles. (**a**) Levy plot for in vitro time scaling. (**b**) Nonlinear regression line for percent absorbed in vivo versus percent released in vitro. (**c**) IVIVC-predicted (lines) versus observed (dots) in vivo percent absorbed.

The in vitro release profiles were generated using an accelerated assay, leading to a faster TV-46000 degradation than observed in vivo based on the deconvoluted in vivo absorption profiles. Therefore, the obtained in vitro release profiles needed to be scaled in time prior to the correlation step with the deconvoluted profiles. This task was performed by linear interpolation using the Phoenix IVIVC Toolkit. The resulting Levy plot is shown in Figure 6a. The linear regression line shown in Figure 6a showed that the fit was not optimal (R^2^ = 0.91) due to large differences between dose strengths. A dedicated time scaling factor would be required for each strength to better fit the data. However, this is not a common practice and due to limited data available at this stage, decision was made to proceed with the presented time scaling. The equation resulting from the linear regression is shown in Equation (3) and was used to scale in vitro time points prior to the correlation step.

Equation (3): Linear regression equation for in vitro time scaling.

(3) TVivo=11.05×TVitro

Following the time scaling step, both the in vitro percent released and the in vivo percent absorbed were on the same time scale, thus enabling us to fit a regression line and obtain the final IVIVC model. Several linear and nonlinear models (Higuchi, Sigmoid, Gompertz, polynomial 2nd order, polynomial 3rd order) have been fitted to the data, and the best results were obtained with the nonlinear polynomial 2nd order model (R^2^ = 0.987) shown in Figure 6b. The equation resulting from the nonlinear regression is shown in Equation (4).

Equation (4): Nonlinear regression equation for IVIVC.

(4) $$\%absorbed=0.0085\times {\left(\%released\right)}^{2}+0.319\times \left(\%released\right)+0.113$$

The obtained IVIVC model (Equation (4)) was used to compute the in vivo percent absorbed values based on in vitro percent released and predicted values were compared with observed ones. As seen in Figure 6c, the predicted profile was very similar to the observed one for the 50 mg dose. However, the terminal portion of the 225 mg profile was slightly overestimated by the model as a result of the lack of fit of the terminal portion observed in the correlation plot.

#### 3.4.3. Convolution and Internal Prediction Errors

The convolution step was performed using the predicted in vivo percent absorbed versus time profiles. For each dose strength, the predicted in vivo percent absorbed profiles were used as a surrogate of the Weibull release function in the respective calibrated PBPK models. The internal validation consisted of computing the percent prediction error (PE) between the predicted exposure parameters (C_max_, AUC_0-t_) and the observed ones for each strength used in the model.

The results of the internal validation are shown in Table 8. The mean prediction errors were 16.7% for C_max_ and 19.4% for AUC_0-t_. In the lack of relevant in vitro data, external validation was not performed.

## 4. Discussion

LAI antipsychotics are gaining prominence as a compelling option for chronic treatment. The effective design of release characteristics for LAIs are typically guided by the optimal drug PK profile identified through the exposure-response relationship and the minimum effective and maximal tolerated concentrations. To streamline the development of LAI products, establishing a pharmacometric framework is crucial. A significant challenge in this endeavor is posed by the highly variable and irregular PK profile exhibited by LAI products. Typically, this profile is comprised an initial release phase in which a small fraction of the drug swiftly transitions from the injection site to systemic circulation, and a main release phase, during which the majority of the active ingredients are released from the injection site into the systemic circulation.

The complexity and multiphase nature of the in vivo drug release process pose challenges for modeling the pharmacokinetic properties of LAI products effectively [19]. Various pharmacokinetic strategies have been developed and applied to analyze atypical absorption profiles, including physiologically based pharmacokinetic approaches, double or triple Weibull in vivo release models, parallel zero-order immediate release followed by first-order release, transit compartments for delayed drug release, a combination of immediate first-order release and transit compartments, and inverse Gaussian density absorption. Recently, a convolution-based modeling approach has proven to be a powerful and flexible tool for modeling complex pharmacokinetics of extended-release and LAI products. This approach maximizes the benefit-risk ratio of a treatment by optimizing drug release properties through IVIVC and integrated PK/pharmacodynamic models.

The modeling strategy presented here consisted of building and calibrating a PBPK model in dogs using the available experimental and literature data, and then to scale it to the human physiology using specific human parameters. To construct a relevant model for TV-46000 in dog, both clearance and the tissue-to-plasma partition coefficient (K_p_) for risperidone were characterized using iv and IR sc data, respectively. The modeling strategy section details the determination of K_p_ for adipose tissue using a calibrated IR sc model. This parameter reflects the distribution of risperidone from the subcutaneous space to the systemic circulation, independent of any formulation-related parameter. It is a pivotal parameter that needs to be accurately estimated in the final PBPK model to make the distinction between the compound-driven and the formulation-driven kinetics. Using the calibrated PBPK model for risperidone sc administration in dog, the TV-46000 release kinetic was characterized by fitting a double-Weibull release function to the dog PK data. Graphical goodness-of-fit and prediction errors around the predicted PK parameters (C_max_ and AUC_0–∞_) attested of the overall good accuracy of the final PBPK model in dog.

Assuming that the K_p_ and the drug release function were similar between dog and human, the PK profiles of risperidone and 9-OH-risperidone were predicted using a new PBPK model adjusted with specific human parameters (B/P ratio, fraction unbound, in vitro metabolism). The simulated TV-46000 PK profile in humans showed that the shape of the predicted risperidone and 9-OH-risperidone PK profiles were different from the observed one, thus suggesting that the TV-46000 release profile was species dependent and cannot be directly extrapolated from dog to human. It is interesting to note that the Weibull parameters were almost identical for the 50 and 225 mg dose levels, showing that the mechanisms of release were preserved in vivo between doses and dose volume. The Weibull parameters indicated that the overall shape of the profiles was similar between doses, but the release was significantly slower for the higher dose. When comparing the two different doses of the drug, the primary difference observed was in the maximum percentage of the drug that was released. Specifically, it indicates that a larger dose volume resulted in a lower total amount of risperidone being released over the same period. This suggests that increasing the volume of the dose may slow down the release rate within a given timeframe.

The IVIVC model was built with only two different strengths, 50 mg and 225 mg which represented two different injection volumes. These doses were strategically chosen to cover the entire range of tested doses for TV-46000, aligning with the approved clinical dosage range. A multi-step approach was developed to leverage the benefit of PBPK modeling for mechanistic deconvolution using the Weibull release function. A time scaling step was introduced but showed some limitation due to differences in time scale between both strengths. Following convolution using the PBPK human model, the internal validation showed promising results. The lack of clear regulatory guidance for IVIVC development of complex injectable formulations makes it difficult to determine the validity of this IVIVC. The only reference to date was the FDA guidance on IVIVC development for extended-release oral dosage forms [20] that recommended an average PE of a maximum 10% to claim a level A IVIVC. While level A IVIVC cannot be claimed here, the presented IVIVC model can still be useful considering that the prediction errors are still lower than the inter-individual variability observed in the clinical data. Improving the overall model accuracy (in particular the time scaling step) and generating in vitro data to run the external validation might increase the chances of seeing such work accepted by the regulatory agencies with proper justification.

## 5. Conclusions

TV-46000 is a novel formulation of risperidone as an extended-release suspension for sc injection. This work presents the strategy and results of the PBPK modeling and IVIVC for the prediction of TV-46000 pharmacokinetic profile in humans using in vitro release, intravenous (iv) and subcutaneous (sc) single dose pharmacokinetic data in beagle dogs. While level A IVIVC cannot be claimed, the presented work combining PBPK and IVIVC modeling represents an interesting alternative approach for complex injectable formulations where classical methods are not applicable. Adding extra strengths and generating in vitro data to perform the external validation would enhance the overall model accuracy, thus improving the chances to establish a successful IVIVC correlation.

## Figures and Tables

**Table 1:** Physicochemical properties of risperidone.

Parameter	Risperidone	9-OH-Risperidone
Molecular weight (g/mol)	410.49	426.48
LogP	3.04	2.32
Solubility (pH 7.4, mg/mL)	0.171	0.171
pKa (Base)	8.24	8.24
pKa (Acid)	3.11	3.11

**Table 2:** Michaelis–Menten constants for risperidone in vitro metabolism.

Enzyme	Vm (pmol/min/pmolCYP)	Km (µM)
CYP2D6 1	13.9	40.4
CYP2D6 2	3.155	0.534
CYP3A4	12.4 ± 0.5	37.4 ± 3.2

Legend: Vm; maximum velocity achieved at maximum substrate concentration, Km; substrate concentration when the reaction velocity is 50% of Vm.

**Table 3:** Summary table of observed vs. predicted PK parameters using the in vivo models in dogs.

Model Parameters	Observed Value	Predicted Value	Error (%)
Calibrated iv model			
C_max_ (ng/mL)	109.1	88.4	18.9
AUC_0–∞_ (h × ng/mL)	115.7	121.7	5.2
Calibrated IR model			
C_max_ (ng/mL)	264.3	234.8	11.1
AUC_0–∞_ (h × ng/mL)	510.6	560.3	9.7
Calibrated LAI sc model			
C_max_ (ng/mL)	12.7	14.2	11.8
AUC_0–∞_ (h × ng/mL)	5552.1	5730.6	3.2

**Table 4:** Optimized double-Weibull parameter values for TV-46000 in dogs (50 mg/dog).

Parameter	Value
Time lag (h)	0
Total released (%)	100
Phase 1—fraction	0.142
Phase 1—time scale	30.88
Phase 1—shape	0.717
Phase 2—fraction	0.858
Phase 2—time scale	6370.7
Phase 2—shape	1.285

**Table 5:** Summary of observed versus predicted PK parameters for risperidone.

Parameter	Observed Value	Predicted Value	Error (%)
C_max_ (ng/mL)	5.0	5.6	12.0
AUC_0–∞_ (h × ng/mL)	4566.7	3196.2	30.0

**Table 6:** Summary of observed versus predicted PK parameters for TV-46000 in humans.

Model Parameters	Observed Value	Predicted Value	Error (%)
TV-46000 50 mg			
C_max_ (ng/mL)	5.05	4.31	13.6
AUC_0–∞_ (h × ng/mL)	4566.7	4414.1	3.3
TV-46000 250 mg			
C_max_ (ng/mL)	8.6	7.8	9.3
AUC_0–∞_ (h × ng/mL)	9365.8	8392.1	10.4

**Table 7:** Optimized triple Weibull parameter values for TV-46000 in humans at 50 and 225 mg.

Parameter	50 mg	225 mg
Time lag (h)	0.090	0.095
Total released (%)	99.75	60.0
Phase 1—fraction	0.197	0.187
Phase 1—time scale	102.1	107.2
Phase 1—shape	0.68	0.65
Phase 2—fraction	0.367	0.367
Phase 2—time scale	2745.8	2745.8
Phase 2—shape	1.32	1.32
Phase 3—fraction	0.436	0.446
Phase 3—time scale	1,823,000	1,914,000
Phase 3—shape	2.05	2.05

**Table 8:** Internal validation of the IVIVC model for TV-46000.

	Strength	Observed	Predicted	Prediction Error (%)
C_max_ (ng/mL)	50 mg	5.0	4.3	14.0
	225 mg	8.7	7.0	19.5
AUC_0-t_ (h × ng/mL)	50 mg	3839	2876	25.0
	225 mg	8296	9440	13.8

## Data Availability

The data sets used and/or analyzed for the studies described in this manuscript are available upon reasonable request.
